# Ecosystem functioning and maximum entropy production: a quantitative test of hypotheses

**DOI:** 10.1098/rstb.2009.0300

**Published:** 2010-05-12

**Authors:** Filip J. R. Meysman, Stijn Bruers

**Affiliations:** 1 Laboratory for Analytical and Environmental Chemistry, Earth System Sciences Research Unit, Vrije Universiteit Brussel (VUB), Pleinlaan 2, 1050 Brussel, Belgium; 2 Netherlands Institute of Ecology (NIOO-KNAW), Korringaweg 7, 4401 NT Yerseke, The Netherlands; 3 Instituut voor Theoretische Fysica, Katholieke Universiteit Leuven, Celestijnenlaan 200D, 3001 Leuven, Belgium

**Keywords:** ecosystems, thermodynamics, entropy production, ecological goal function, food webs

## Abstract

The idea that entropy production puts a constraint on ecosystem functioning is quite popular in ecological thermodynamics. Yet, until now, such claims have received little quantitative verification. Here, we examine three ‘entropy production’ hypotheses that have been forwarded in the past. The first states that increased entropy production serves as a fingerprint of living systems. The other two hypotheses invoke stronger constraints. The state selection hypothesis states that when a system can attain multiple steady states, the stable state will show the highest entropy production rate. The gradient response principle requires that when the thermodynamic gradient increases, the system's new stable state should always be accompanied by a higher entropy production rate. We test these three hypotheses by applying them to a set of conventional food web models. Each time, we calculate the entropy production rate associated with the stable state of the ecosystem. This analysis shows that the first hypothesis holds for all the food webs tested: the living state shows always an increased entropy production over the abiotic state. In contrast, the state selection and gradient response hypotheses break down when the food web incorporates more than one trophic level, indicating that they are not generally valid.

## 1. Introduction: living systems and entropy production

In his classical essay ‘What is life?’, Schrödinger (1944) posed the question of how to reconcile biological organization with the second law of thermodynamics. Schrödinger noted that at first sight, biological systems seem to defy the second law, which dictates that a closed system inescapably moves towards a ‘disordered’ state of maximum entropy. Nonetheless, living systems (cells, organisms, populations and ecosystems) are highly organized, and so how do they generate and propagate such organization? Schrödinger resolved the apparent contradiction by noting that living systems are necessarily open. But openness in itself is not enough. Not all open systems display structure through self-organization. Schrödinger (1944) went further and put forward the vital insight that biological systems can only maintain their internal ‘order’ at the expense of a continuous creation of ‘disorder’ in the external environment through metabolic activity. Later on, Schrödinger's idea was more generally reformulated as that biological systems can maintain a far-from-equilibrium state only through a continuous exchange of energy and matter with their environment, a process that is necessarily accompanied by entropy production (Morowitz 1968).

**Figure 1.**
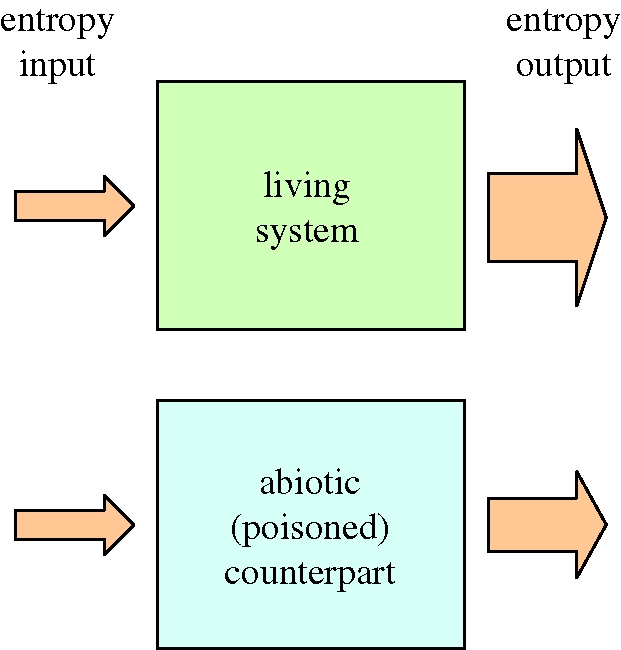
Schrödinger's idea on the thermodynamic fingerprint of living systems. Given exactly the same boundary conditions, a living system should show an increased entropy production (and hence an increased entropy output given the same entropy input). The abiotic counterpart can be thought of as the living system treated with a suitable poison as to prohibit all biological activity.

Schrödinger's analysis invoked a shift in the mechanistic thinking about biological systems and gave the debate a strong thermodynamic imprint. Starting from the observation that biological organization can only build and maintain its structure by grace of enhanced entropy production, Ulanowicz & Hannon (1987) rephrased and sharpened Schrödinger's idea, proposing that increased entropy production effectively serves as a fingerprint of life. Living communities augment the rate of entropy production over what would be found in the absence of biota, all other things being equal. To illustrate this idea, consider the simple scheme in figure 1, which shows a living system and its non-living counterpart under identical boundary conditions. Two flow-through systems are driven by the same input—think of two chemostat reactors that are fed by the same aqueous solution, which contains some (food) substrate compound. In the first reactor, some appropriate poison has been added to the inflow so that only abiotic chemical reactions are converting the substrate. In contrast, the second reactor has been inoculated with a sufficiently rich microbial culture, and so a community of micro-organisms is thriving on the food substrate inside the reactor. Ulanowicz & Hannon (1987) then imply that the microbial population in the second reactor needs to generate additional entropy in order to ‘make a living’. Accordingly, if one would measure and compare the overall entropy production in the two systems, the second reactor would show the highest entropy production rate, and this difference could be interpreted as a sign of life.

In the discussion on the relation between functioning of living systems and entropy production, a new aspect has been added in recent years: the notion of maximizing the entropy production. The general idea is that complex systems somehow self-organize so as to reach a state in which they produce entropy at the maximum possible rate, given the prevailing constraints. This hypothesis of ‘maximal entropy production’ (MEP) is not restricted to ecology or ecosystem research, but has surfaced independently under various forms in a range of disciplines—see reviews by Martyushev & Selezvev (2006) and Kleidon (2009) and the other contributions in this special issue. There are, however, many ways in which ‘maximization’ can be interpreted, and there are different ways in which the ‘entropy production rate’ is defined (depending on which processes one accounts for). As a result, the MEP hypothesis should not be regarded a single ‘principle of nature’, but rather as a class of hypotheses, some of which appear only weakly connected (if not entirely unrelated). A clear challenge is to disentangle these different MEP interpretations, though this is beyond our scope here. Here, we mainly restrict ourselves to the investigation of MEP ideas in ecology or ecosystem research (although some of our results should have implications outside ecology).

But also in the ecological literature, there is scope for confusion. In addition to MEP, the maximization idea has been expressed in many ways, using a variety of terminology, such as ‘maximal gradient destruction’, ‘maximum energy dissipation’ and ‘maximum energy destruction’ (Schneider & Kay 1994; Fath *et al.* 2001; Jørgensen & Svirezhev 2004; Schneider & Sagan 2005; Aoki 2006). When making abstraction of the loose terminology and restricting ourselves to the standard concepts of non-equilibrium thermodynamics (that is, expressing everything in terms of entropy production), there appears nonetheless to be a common ground, which can be illustrated by our example in figure 1. The MEP prediction is that the living system will not only have a higher entropy production than the non-living one, but also that the living system will self-organize itself so that its entropy production is maximized in some way (there are different ways in which maximization can be interpreted, as shown below). In other words, the MEP hypothesis imposes stronger constraints than just increased entropy production over the abiotic state. Instead, the entropy production rate is thought to act as a sort of goal function, i.e. an extremal principle that is relevant for the development and operation of living systems.

Until now, neither the idea of ‘increased entropy production as a sign of life’, nor the idea that ‘living systems maximize their entropy production’ has been truly quantitatively tested. This is exactly the purpose of the present paper. Our goal is to investigate the link between entropy production and ecosystem functioning in a quantitative way. The approach taken is rather straightforward: (i) construct a set of archetypal ecosystem models, which are well known from ecological theory (resource–consumer, resource–consumer–predator, resource–consumer–omnivore), (ii) calculate the associated entropy production within these ecosystem models, and (iii) verify whether Schrödinger's idea (does the ecosystem exhibit an increased entropy production when compared with the non-living system?) or the MEP hypothesis (does the ecosystem poise itself in a state of MEP?) hold.

The paper is organized as follows. In the first section, we clarify what is meant by the constraint of MEP and show that there are (at least) two different ways in which this constraint can be interpreted. Subsequently, we detail the formulation of the different food web (ecosystem) models that are used in the simulations. In the final section, we then present the results of our thermodynamic analysis of these various ecosystem models, verifying whether the various hypotheses with regard to the entropy production do hold or not.

## 2. State selection and gradient response principles

Schrödinger's basic idea was that biological organization requires increased energy production. However, his reasoning was not complete. Schrödinger did not provide a mechanistic explanation of how the observed self-organization was actually connected to the increased entropy production. This link was only later investigated, most prominently in the work of Ilya Prigogine and co-workers. They refined Schrödinger's order-from-disorder idea and developed it into a more general theory on self-organization in far-from-equilibrium systems (Prigogine 1967; Glansdorff & Prigogine 1971; Nicolis & Prigogine 1977).

**Figure 2.**
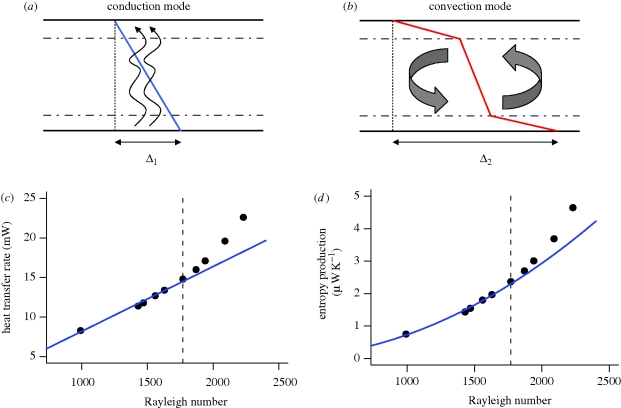
In a Rayleigh–Bénard experiment, a viscous liquid is confined between two plates and heated from below. The temperature difference across the plates forms a measure of how far the system is displaced from equilibrium and is referred to as the thermodynamic gradient (expressed by the dimensionless Rayleigh number Ra). (*a*) When the thermodynamic gradient remains small (*Δ*_1_), heat passes through the liquid by thermal conduction (blue linear temperature profile). (*b*) When the heating is intensified (*Δ*_2_), a regular pattern of hexagonal convection cells appears once beyond a certain critical threshold of the thermodynamic gradient (red temperature profile with boundary layers). Schneider & Kay (1994) used the (*c*) and (*d*) graphs to forward two principles that govern the functioning of complex dissipative systems: state selection and gradient response (see text). (*c*) The heat transfer is plotted as a function of the thermodynamic gradient (Ra). (*d*) The entropy production is plotted as a function of the thermodynamic gradient (Ra). Data points (black solid markers) are experimental data from Silveston (1958). The blue line shows the response if thermal conduction only would be acting. Beyond a given critical Rayleigh number, Rayleigh–Bénard convection sets in, and the data deviate from the thermal conduction response.

When open systems are subject to a sufficiently large thermodynamic gradient (like a temperature, velocity or concentration difference), it is observed that self-organized structures can spontaneously emerge. The archetypal example of this phenomenon is Rayleigh–Bénard convection (Bénard 1901; Rayleigh 1916). In a Rayleigh–Bénard experiment, a shallow layer of a viscous fluid is heated from below (figure 2). When the temperature difference across the fluid is small, heat transfer solely occurs through thermal conduction. Once beyond a critical temperature difference, a regular pattern of convection cells emerges, resulting in increased heat transfer across the fluid (Koschmieder 1993; Manneville 2006).

Although the phenomenon of Rayleigh–Bénard convection was already known from the start of the twentieth century, the work by Prigogine and co-workers established a crucial link between self-organization and entropy production. They stated that the formation of ordered structures in open systems involves a threshold phenomenon, which is marked by increased ‘dissipation’; hence they coined the term ‘dissipative structure’ (Nicolis & Prigogine 1977). In this, the term dissipation acts as a synonym for entropy production. In the Rayleigh–Bénard case, the convection cells emerge when temperature difference across the plates increases beyond a critical value. In this convective regime, the heat transfer and the entropy production are greater than if only conduction would be acting (figure 2*c*). Although Rayleigh–Bénard convection is the best-known example, similar threshold phenomena give rise to vortices in Taylor–Couette flow (Koschmieder 1993) and occurs naturally under the form of tornadoes and hurricanes (Emanuel 1999).

Prigogine's (physical) concept of a ‘dissipative structure’ was eventually picked up in the ecological literature. Particularly the example of Rayleigh–Bénard convection served as a source of inspiration to formulate new (and radical) hypotheses about the thermodynamic functioning of biological systems (Swenson 1989; Schneider & Kay 1994). A quite influential paper in this respect was Schneider & Kay (1994), that propagated the captivating but audacious idea that Rayleigh–Bénard convection could serve as a blueprint for all self-organized systems, including all living matter ‘ranging from cells to ecosystems’ (see Schneider & Sagan 2005 for an updated version of this idea). Note the drastic character of this ‘universal self-organization’ hypothesis: the functioning of all self-organized non-equilibrium systems, both living and non-living, should obey the same principles and mechanisms. Nonetheless, with little reservation, Schneider & Kay (1994) referred to their idea as a ‘restated second law’ and claimed that it provided ‘a thermodynamically consistent explanation of why there is life, including the origin of life, biological growth, the development of ecosystems and patterns of biological evolution observed in the fossil record’ (Schneider & Kay 1994).

Although their starting idea was intriguing and original, the actual treatment by Schneider & Kay (1994) was exclusively verbal, lacked quantitative rigor and was not presented in terms of standard thermodynamic concepts (like entropy or free energy). Overall, they summarized their hypothesis as that ‘all living entities will take advantage of all available means to counter externally applied gradients’. In later writings, this was even more loosely rephrased as that ‘nature abhors a gradient’ (Schneider & Sagan 2005). Unfortunately, such vague formulations invoke all sorts of interpretation issues and do not allow any quantitative testing.

However, as we show here, the underlying premise that Rayleigh–Bénard convection serves as a ‘universal blueprint’ for self-organized systems can be readily translated into testable criteria. Two central graphs in this argument are the bifurcation plots for the heat transfer and entropy production in a Rayleigh–Bénard system, as reproduced in figure 2*c*. If all forms of self-organization act like Rayleigh–Bénard convection, as was speculated by Schneider & Kay (1994), then we can deduce two principles that should govern the entropy production in complex self-organized systems (including ecosystems):

— The *state selection principle* details how the system will behave under constant external boundary conditions. When a system can attain multiple steady states, the stable state will be the one that shows the highest entropy production rate.
— The *gradient response principle* details how the system will behave when the external boundary conditions are changed. When the thermodynamic gradient increases, the system's new stable state should be accompanied by a higher entropy production rate.

Note the drastic generalization that underlies this reasoning. One particular physical example of a dissipative structure (Rayleigh–Bénard convection near the critical bifurcation point) is used as a template to explain the functioning of all biological organization, ranging from cells to ecosystems. The state selection and gradient response principles can be regarded as two different interpretations of the MEP hypothesis. The state selection principle makes connection between entropy production and the stability of steady states and embodies a true maximization. When comparing the entropy production rate of a series of possible steady states, the maximum entropy production rate in the set should correspond to the stable state. This state selection interpretation of MEP has also been advanced outside ecology: Shimokawa & Ozawa (2001) found that in ocean general circulation models, the stable solution corresponds to the one showing the highest entropy production rate. The gradient response principle provides a different constraint, which does not strictly involve a maximization and hence has a looser connection to MEP. When the thermodynamic gradient increases, the new stable state of the system should only display a higher entropy production rate. In the next section, we will specify a number of food web models, in order to verify whether these presumed principles hold for ecosystems.

## 3. Ecosystem model formulation

The ecosystem models that are analysed here form an idealization of a detrital-based heterotrophic ecosystem, as found in ocean sediments and also terrestrial soils (the parameter set is based on deep sea sediments). This detrital-based ecosystem has the advantage that its ecological and thermodynamic descriptions can be linked in a straightforward fashion. On the one hand, the ecosystem can be described by the conventional equations of food web ecology (Armstrong & McGehee 1980; Tilman 1982; Loreau & Holt 2004). On the other hand, the food web interactions can all be cast into chemical reaction equations, which then can be treated with the standard expressions of chemical thermodynamics (e.g. Kondepudi & Prigogine 1998). This way, we can directly employ the theory of chemical thermodynamics to calculate the entropy production associated with ecological interactions (see Meysman & Bruers 2007 for a detailed thermodynamic analysis of detrital-based ecosystems).

### (a) Metabolic transformations

The model presented here describes a simplified food web in the subsurface ecosystem of marine sediments. This system is fuelled by detrital organic matter that is fixed by photosynthesis within the upper ocean, settles through the water column and rains down on the sediment surface. Consider a consumer that feeds on this organic resource, converting it into biomass and metabolic end products (e.g. a population of heterotrophic bacteria feeding on detritus). The associated metabolic transformation can be represented by the reaction equation3.1
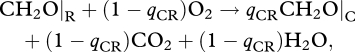

where CH_2_O|_R_ and CH_2_O|_C_, respectively, represent the stoichiometry of resource and consumer. This transformation describes in a simple manner the coupling of anabolism (biomass synthesis) to catabolism (respiration). The yield factor *q*_CR_ represents the amount of consumer biomass that results from the assimilation of one unit of resource. Or equally, 1 − *q*_CR_ denotes the respiration cost associated with the biomass synthesis. Our model assumes that the consumer's growth is only limited by the organic carbon resource (no other nutrients are limiting). Accordingly, the metabolic transformation is not constrained by the availability of the electron acceptor (O_2_), and as a result, we can rewrite the above reaction equation in a simplified form as follows:3.2
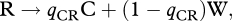

where R symbolizes the organic resource, C the consumer biomass and W the metabolic ‘waste’ product CO_2_. This reaction equation hence specifically focuses on the carbon transformations associated with consumer metabolism.

The consumer C itself is preyed upon by a predator P. To make this interaction more general, we allow the predator also to directly feed on the resource (Holt & Polis 1997; Mylius *et al.* 2001). The predator thus becomes an omnivore. Adopting the same reasoning as above, the predator's metabolism can be represented by the simplified reaction equations3.3
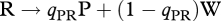

and3.4
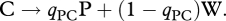

The first equation models the predator feeding on the resource. The second equation models the predation on the consumer.

In our idealized model description, the biomass of the consumer and predator are simply assembled from elementary building blocks of the resource R. When an organism suffers a natural death (from accident, disease or ageing, though excluding predation), biomass disassembles back into these basic resource units. This turnover of biomass is described via the reaction equations3.5
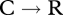

and3.6
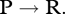

These processes essentially represent the internal recycling within a detrital-based food web and lead to closure of the food web mass balances. The resource that is ‘recycled’ becomes again available for biomass synthesis or respiration.

The set of transformations as detailed above would be the selection as incorporated in a traditional resource-competition model from theoretical ecology (Grover 1997; Loreau & Holt 2004). Here, however, to ensure thermodynamic consistency, we need to add one more process: an abiotic pathway of resource conversion. In the above metabolic transformations, the respiration of the consumer and predator convert the organic resource into the waste product CO_2_. In addition to this biological respiration, we now include the abiotic oxidation pathway3.7
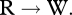

This abiotic oxidation represents a slow, chemical mechanism that is always present in the background (think of the slow chemical oxidation rate of organic matter that would be measured when the soil or sediment is treated by a biocide killing off all biological activity). Under natural conditions, this strictly chemical pathway will be far exceeded by the biotic respiration rate, and hence it will be quantitatively negligible. However, its inclusion in the model is qualitatively important. As shown below, abiotic oxidation functions as the ‘incoherent’ dissipation mode in our ecosystem model and thus forms the analogue of thermal conduction in the Rayleigh–Bénard set-up.

### (b) Mass balances

**Figure 3.**
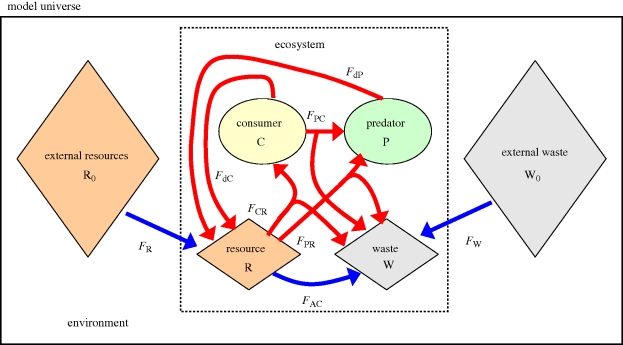
Flow diagram of the simplified ecosystem model. Blue arrows indicate abiotically controlled flows. Red arrows indicate biologically mediated flows. The external compartments are considered ‘infinite reservoirs’, so that their concentrations R_0_ and W_0_ would remain fixed. Table 1 provides the constitutive expressions for the flows.

The flow scheme in figure 3 summarizes our model statement of the carbon dynamics within the ocean floor ecosystem. It incorporates two compartments, termed ‘ecosystem’ and ‘environment’. Both ecosystem and environment contain one resource (R) and one waste (W) reservoir. In addition, the ecosystem also contains the consumer (C) and predator (P) biomass reservoirs. The principal difference between the R and W reservoirs in ecosystem and environment is their size. The external reservoirs are considered ‘infinite’, so that the concentrations 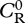 and 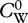 are fixed model parameters (instead of state variables). The resulting exchange between ecosystem and environment is represented by the flows *F*_R_ and *F*_W_. Accounting for all the above transformations, the ecosystem model thus consists of following mass balances:
3.8
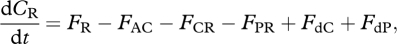

3.9
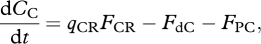

3.10
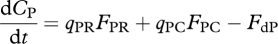
and
3.11
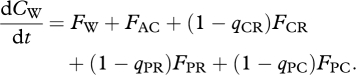

The symbols *C*_*i*_ represent the carbon concentrations in each of the reservoirs. This equation set is very similar to that of a conventional ecosystem model. The only small differences are that we include the abiotic oxidation mechanism (as already discussed), and that we explicitly include the metabolic waste product W as a state variable. As shown below, the waste concentration is explicitly needed to calculate the entropy production associated with resource conversion.

If one looks only at the input and output, the net transformation that occurs within the ecosystem consists of the oxidation of organic resource:3.12
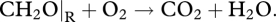

This reaction effectively symbolizes the core dissipative process within our ecosystem: high-quality resources (CH_2_O, O_2_) are converted into low-grade waste products (CO_2_, H_2_O). The associated reaction rate is termed the ecosystem metabolism:3.13
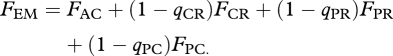

This flow contains both an abiotic component (*F*_AC_) as well as factors due to the biotic respiration of consumers and predators (the remaining terms). The ecosystem metabolism *F*_EM_ will play a crucial role in our thermodynamic analysis, as shown below.

The constitutive expressions for all the flows are summarized in table 1. The exchange with the environment is modelled by a linear expression as is usually done in ecological models (Tilman 1982; Loreau & Holt 2004). The associated transport coefficients can be directly calculated from the dominant physical transport processes in the system, as detailed in Meysman & Bruers (2007) (see also the electronic supplementary material). The resource uptake by the consumer and predator is modelled by a classical Holling type I functional response. The abiotic conversion of resource is described by a standard first-order kinetic rate expression. Rather than the usual dependency on *C*_R_, these expressions feature the term C_R_ − *C*_W_/*K*_eq_, where the quantity *K*_eq_ denotes the thermodynamic equilibrium constant for the oxidation reaction (3.12). This modification is simply implemented for thermodynamic consistency: the transformation of resource—both biotic and abiotic—should vanish in the thermodynamic equilibrium. The feeding of predator on the consumer is also modelled by Holling type I functional response. Note that one could implement more complex functional responses for the biotic interactions. Such descriptions are, however, not explored here, as they provide qualitatively similar results, but mathematically they lead to more intricate expressions.

### (c) Entropy production

For our analysis, we need to calculate the total entropy production *σ*_tot_ that takes place in our model ‘universe’. Formally, we can decompose the total entropy in our set-up into separate contributions of ecosystem (‘sys’) and environment (‘env’), i.e. *S*_uni_ = *S*_env_ + *S*_sys_. The resulting entropy balance hence becomes3.14
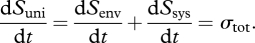

Because our model universe is isolated as a whole, no entropy transfer takes place across its boundaries, and so the left-hand side of equation (3.14) only features the ‘internal’ entropy generation *σ*_tot_. This quantity is obtained by summation of the contributions of the individual flows3.15
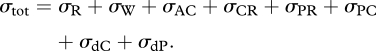

Together, these eight individual entropy production rates quantify all ‘dissipation’ that occurs within our model universe, where the term *dissipation* is synonymous with entropy production. The second law of thermodynamics requires that for each independent flow *F*_*i*_, the associated entropy production *σ*_*i*_ should be positive (Nicolis & Prigogine 1977; Kondepudi & Prigogine 1998). A given flow is ‘independent’ when it is not coupled to any of the other flows. In our model, all eight flows are independent, and so the associated entropy production rates should all be positive, *σ*_*i*_ > 0. As a consequence, we directly find that *σ*_tot_ > 0. The entropy of our model universe as a whole thus can only increase, in accordance with the second law statement for isolated systems.

In non-equilibrium thermodynamics, the entropy production *σ*_*i*_ associated with a given flow *F*_*i*_ is calculated as the product of that flow with a corresponding thermodynamical force *X*_*i*_ (Nicolis & Prigogine 1977; Kondepudi & Prigogine 1998):3.16
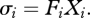

The expressions for the flows *F*_*i*_ were already listed in table 1. The expressions for the associated forces *X*_*i*_ are listed along side. The method for calculating these *X*_*i*_ terms is described in detail in Meysman & Bruers (2007). The resource and waste exchange essentially describe a mixing process between two reservoirs A and B at different concentrations. The associated thermodynamic force can be directly calculated as *X*_mix_ = −*ΔG*_mix_/*T* = (*μ*_A_ −*μ*_B_)/*T*, where *ΔG*_mix_ is the Gibbs free energy of mixing, *T* the temperature and *μ* the chemical potential (Kondepudi & Prigogine 1998). All the other flows are, in essence, rates associated with a given chemical reaction. The thermodynamic force associated with a chemical reaction is calculated as *X*_reac_ = −*ΔG*_reac_/*T*, where the Gibbs free energy *ΔG*_reac_ denotes the difference in chemical potential between reaction products and reactants (Kondepudi & Prigogine 1998). For the abiotic compounds R and W, we adopt ideal behaviour, so the chemical potential scales with the logarithm of the concentration, i.e. *μ* = *μ*^ref^ + *RT* ln(*C*/*C*^ref^), where *R* denotes the universal gas constant and the concentration *C*^ref^ refers to some reference state. However, there is presently no theoretical approach that allows to calculate the chemical potential of the biotic compounds C and P—see discussion on ‘How to calculate the chemical potential of a rabbit’ in Meysman & Bruers (2007). Fortunately, we can circumvent this problem by considering only the steady-state situation. In the steady state, the chemical potentials of the biotic compounds will drop from the equations (see below).

**Table 1.** Summary of the rate expressions *F*_*i*_ (second column) and the associated thermodynamic forces *X*_*i*_ (third column) that are used in the ecosystem model. The flows *F*_*i*_ are specified in figure 3. The symbol *T* is the temperature, *R* denotes the gas constant and *μ*_*i*_ are chemical potentials.

transformation	constitutive equation	thermodynamic force
C–R interaction	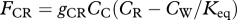	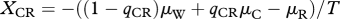
P–R interaction	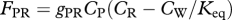	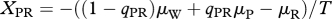
P–C interaction	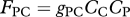	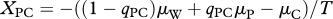
C turnover	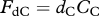	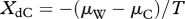
P turnover	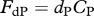	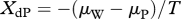
abiotic oxidation	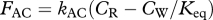	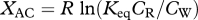
R exchange	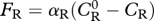	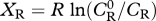
W exchange	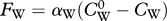	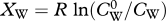

### (d) Steady state analysis

For fixed boundary conditions (i.e. a set of fixed values for 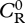 and 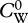), we can show that the ecosystem model will always reach a steady state, thus excluding the possibility of oscillatory and chaotic dynamics (see the electronic supplementary material). Note that the term ‘steady state’ should be interpreted with caution: the time invariance only applies to the ecosystem, not the environment. The assumption that 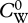 and 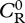 are fixed is nothing but a suitable approximation for large reservoirs with a slow relaxation time. In actual fact, the environment cannot reside in a steady state, as can be seen from the entropy balance (3.14) of the total set-up. When applying the steady-state condition, only the ecosystem term d*S*_sys_/d*t* vanishes, and so one obtains3.17
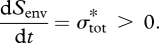

To make a distinction with transient properties, steady-state values are denoted by an asterisk superscript *. In the steady state, all entropy that is generated within the set-up will be transported to the environment and accumulate there. This illustrates that in the steady state, the properties of the environment do *not* remain constant in time.

For the ‘ecosystem’ compartment, the steady-state condition requires that its four state variables should no longer vary with time. For both the consumer and predator, this implies that the rate of biomass synthesis should match the turnover rate. Because there is no net biomass accumulation within the food web, the ‘ecosystem metabolism’ (i.e. the total production of 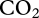) should match the output of waste, which in its turn should match the input of resource:3.18
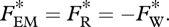

Also, because of the various interdependencies between the flows in the steady state, one can easily show that the total entropy production reduces to3.19
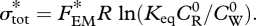



This expression does no longer contain the ‘unknown’ chemical potentials of the biotic compounds C and P. Accordingly, we have circumvented the previously discussed difficulty of defining the chemical potential of biomass (Meysman & Bruers 2007). The total entropy production 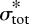 depends on the external boundary conditions imposed upon the ecosystem (via 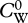 and 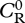) and on the ecosystem metabolism of the food web (via 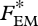).

### (e) Simulations

In a Rayleigh–Bénard experiment, the system is driven further out of equilibrium by increasing the temperature difference *Δ* = *T*_H_ − *T*_L_. In the parlance of non-equilibrium thermodynamics, the quantity *Δ* is referred to as ‘thermodynamic gradient’ that is imposed upon the system. When *Δ* = 0, the set-up resides in thermal equilibrium and no heat transfer takes places across the fluid layer. If one subsequently increases *Δ* > 0, then, first, heat transfer starts by conduction, but once the thermodynamic gradient passes a critical value *Δ* > *Δ*_*c*_, convection cells emerge and heat transfer is dominated by convection.

Here, we will simulate a similar experiment in our ecosystem models. In our case, the basic transformation that occurs within the ecosystem is the oxidation of resource R into waste W as given by reaction (3.12). Accordingly, the thermodynamic gradient is theoretically the difference in chemical potential 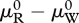 between the reservoirs in the external environment. Instead of using chemical potentials, we opt for a pragmatic but equivalent alternative and express the thermodynamic gradient as the concentration difference imposed by the environment as3.20
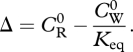

When the thermodynamic gradient vanishes, i.e. 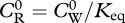, the environment itself resides in a state of chemical equilibrium. In this ‘dead state’, no conversion of resource into waste products can take place—neither biotic nor abiotic. In our simulations, we will gradually increase the thermodynamic gradient *Δ* that is imposed upon our ecosystem model and observe what happens.

All simulations were performed with a representative set of parameters for a deep sea sediment ecosystem. The parameter values are discussed in more detail in the electronic supplementary material and are summarized in table 2. Three different food web models are examined: (A) a simple resource–consumer interaction (*g*_PR_ = *g*_PC_ = 0), (B) a resource–consumer–predator interaction (*g*_PR_ = 0; *g*_PC_ as in table 2), and (C) a resource–consumer–omnivore interaction (*g*_PR_ and *g*_PC_ as in table 2). For each value of *Δ* > 0, we calculated the steady-state values of the ecosystem concentrations (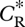, 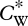, 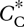, 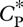) and assessed their linear stability. These calculations are summarized in the electronic supplementary material, and their results are fully consistent with previous stability analysis of omnivory models (Mylius *et al.* 2001). From the ecosystem concentrations, one can calculate directly the ecosystem metabolism (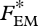), and the total entropy production (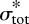). This way, we are able to assess the validity of the entropy principles discussed in the introduction.

**Table 2.** Summary of the parameter values employed in our ecosystem model for a deep sea sediment. The specification of these parameter values is discussed in more detail in the electronic supplementary material. Values are based on the references.

parameter	symbol	unit	value	reference
temperature	*T*	K	283	
gas constant	*R*	J mol^−1^ K^−1^	8.3143	
resource exchange	*α*_R_	yr^−1^	0.01	Middelburg *et al.* (1997), Boudreau (1998)
waste exchange	*α*_W_	yr^−1^	10	Boudreau (1997, 1998)
abiotic conversion	*k*_AC_	yr^−1^	0.01	
external resource	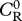	kmol C m^−3^	0.01–20	Middelburg *et al.* (1997), Andersson *et al.* (2003)
external waste	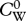	mol C m^−3^	2.5	Sarmiento & Gruber (2006)
equilibrium constant	*K*_eq_	—	250	
growth rate C on R	*g*_CR_	(mol C)^−1^ yr^−1^	0.05	del Giorgio & Cole (1998), Dixon & Turley (2001)
yield factor C on R	*q*_CR_	—	0.2	del Giorgio & Cole (1998)
turn-over rate C	*d*_C_	yr^−1^	10	Dixon & Turley (2001)
growth rate P on R	*g*_PR_	(mol C)^−1^ yr^−1^	0.01	
yield factor P on R	*q*_PR_	—	0.2	
growth rate P on C	*g*_PC_	(mol C)^−1^ yr^−1^	30	
yield factor P on C	*q*_PC_	—	0.2	
turn-over rate P	*d*_P_	yr^−1^	10	

## 4. Results and analysis

### (a) Resource–consumer

**Figure 4.**
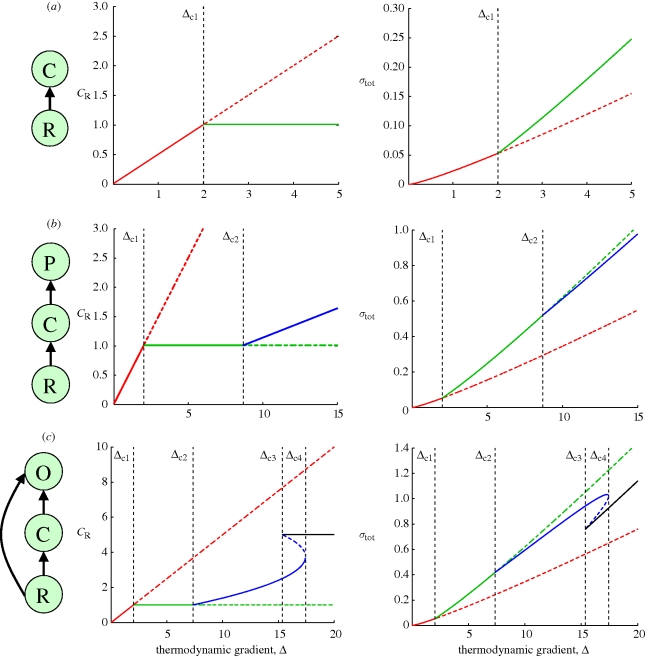
Bifurcation plots of the resource concentration CR (left panels) and entropy production *σ*_tot_ (right panels) for three elementary food webs. Black arrows denote trophic links between food web compartments (R, resource; C, consumer; P, predator; O, omnivore). Steady-state values are plotted as a function of the food supply (more precisely quantified as the thermodynamic gradient *Δ* = *R*_0_ − *W*_0_ /*K*_eq_). Full lines represent stable states. Dashed lines represent unstable states. (*a*) model A: resource–consumer; (*b*) model B: resource–consumer–predator; and (*c*) model C: resource–consumer–omnivore.

Note that model A hardly deserves the qualification ‘food web’ as it only contains a single consumer compartment (one can think of a single group of heterotrophic sediment bacteria that are living from the incoming organic matter). Still, the simulation results of model A are very instructive. When plotting the ecosystem metabolism (figure S1, electronic supplementary material) and the total entropy production rate (figure 4*a*) as a function of the increasing thermodynamic gradient, these graphs are strikingly similar to those of a Rayleigh–Bénard experiment, as shown in figure 2*c*. When *Δ* is small, the thermodynamic gradient is too low to sustain a viable consumer population. The ecosystem metabolism is now only due to the abiotic conversion of resources; this is the analogue of thermal conduction in the Rayleigh–Bénard set-up. When the thermodynamic gradient increases beyond the critical threshold value *Δ*_c1_, a bifurcation occurs. When the system is now seeded with a few consumer organisms (a small ‘biological’ fluctuation), they will be able to establish a stable consumer population. The biotic pathway of resource conversion kicks, and the ecosystem metabolism increases. This bifurcation behaviour is directly analogous to that observed in the Rayleigh–Bénard set-up. In the latter case, small fluctuations in the temperature of the fluid will give rise to the emergence of thermal convection cells, increasing the heat transfer.

The entropy production curve in figure 4*a* shows that the idea of Ulanowicz & Hannon (1987) about ‘increased entropy production as a fingerprint of life’ holds. Given the same boundary conditions (i.e. the same thermodynamic gradient *Δ*), the entropy production of the living system always exceeds that of its abiotic counterpart. If the living system would be suitably poisoned, the ecosystem's operation point would fall back from the branch with the higher entropy production (where the ecosystem metabolism results from both biotic respiration and abiotic oxidation) to the branch with the lower entropy production (where the ecosystem metabolism is only due to abiotic oxidation). In a similar fashion, we can conclude that the state selection and the gradient response principles also hold for the single-consumer food web (A). The stable state of the ecosystem is always associated with the highest entropy production rate (the state selection principle holds). Equally, the entropy production rate of the stable state always increases with increasing resource supply, i.e. 
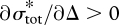
 (the gradient response principle holds).

### (b) Resource–consumer–predator

However, the situation changes when the food web becomes more complex, and more than one trophic compartment is included in the food web. In the output of resource–consumer–predator model B, two bifurcation points are present (Smith & Waltman 1995). Before the first bifurcation point (*Δ* ≤ *Δ*_c1_), only abiotic conversion takes place. Between the first and second bifurcation points (*Δ*_c1_ ≤ *Δ* ≤ *Δ*_c2_), only the consumer is present. When moving beyond the second bifurcation point (*Δ*_c2_ ≤ *Δ*), the resource supply is sufficiently high for the consumer and predator to coexist. Over this whole range, the idea of Ulanowicz & Hannon (1987) holds true: the entropy production of the living system always exceeds that of an abiotic ‘appropriately poisoned’ counterpart. However, the state selection principle clearly breaks down. In the region where consumer and predator coexist, the stable state is no longer associated with the highest entropy production rate, and so the state selection principle no longer holds. In contrast, the gradient response principle still holds in the food web model B: the entropy production rate of the stable state always increases with increasing resource supply, i.e. 
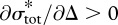
.

### (c) Resource–consumer–omnivore

Finally, in the resource–consumer–omnivore food web (C), the gradient response principle breaks down alongside the state selection principle. In this case, up to four bifurcation points can be present, as known from previous model studies on omnivory (e.g. Mylius *et al.* 2001). Between the third and fourth bifurcation point (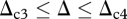), the ecosystem model shows bistability. Both the omnivore-only situation as well as the coexistence of the consumer and omnivore form alternative stable states (the state that is actually attained depends on the initial conditions). Figure 4*c* shows that because of the bistability, the total entropy production rate goes through a local maximum within this interval. Accordingly, the entropy production drops when the thermodynamic gradient increases, and so the gradient response principle no longer holds. Note, however, that the idea of Ulanowicz & Hannon (1987) still holds true over the whole range: the entropy production of the living system always exceeds that of its abiotic counterpart. The operation point with either omnivore and consumer, or the omnivore alone will always exceed the dashed red line (indicating the situation if the ecosystem metabolism would only be due to abiotic oxidation).

## 5. Discussion: ecosystems as dissipative structures?

The concept of a ‘dissipative structure’ emerged from the studies on non-equilibrium thermodynamics by Prigogine and co-workers (Prigogine 1967; Nicolis & Prigogine 1977). The archetypal example of such a dissipative structure, which features in nearly all textbooks on the non-equilibrium physics of fluids, is Rayleigh–Bénard convection (Nicolis & Prigogine 1977). When a thin layer of fluid is exposed to a sufficiently high temperature difference, self-organized structures will emerge that increase the dissipation (i.e. increase the heat transfer and associated entropy production). Schneider & Kay (1994) used the phenomenon of Rayleigh–Bénard convection as a starting point to make a strong and speculative extrapolation: they claimed that the thermodynamic behaviour of Rayleigh–Bénard convection near its bifurcation point serves as a universal template for self-organizing systems. In other words, the audacious proposition was that all self-organizing systems, ranging from ‘primitive physical systems to complex living systems’, operate in a similar fashion as Rayleigh–Bénard convection.

Here, we have tested whether this hypothesis holds true for one particular type of living systems: ecosystems. To this end, we analysed a basic set of food web models, which are standard and well-recognized tools in theoretical ecology (Tilman 1982; Grover 1997). This analysis shows that the analogy between Rayleigh–Bénard convection and ecosystem functioning as proposed by Schneider & Kay (1994) holds only to a limited degree. As it happens, the analogy works fine for the most primitive food web, where a single consumer feeds on a single abiotic resource. In this case, the ecosystem metabolism (i.e. the total 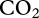 production in the detrital-based ecosystem) clearly forms the counterpart of heat transfer in the Rayleigh–Bénard set-up. The bifurcation plots of the entropy production perfectly match one another (compare figures 2*c* and 4*a*). Accordingly, just like Rayleigh–Bénard convection near the transition point, the establishment of a consumer population feeding on some abiotic resource perfectly fits the ‘dissipative structure’ concept forwarded by Nicolis & Prigogine (1977). When gradually increasing the thermodynamic gradient, such dissipative structures display two regimes, first an ‘incoherent’ baseline regime (thermal conduction and abiotic resource degradation), which then, beyond a critical threshold, shifts to a ‘coherent’ regime (thermal convection and food web metabolism). The hallmark of this ‘coherent’ regime is the spontaneous emergence of structure (convection cells and organism populations) following upon small fluctuations (temperature fluctuations and the seeding of a habitat with a few pioneer organisms). When the coherent regime is stable, the total entropy production rate is always higher than when the associated incoherent regime alone would be present (figures 2*c* and 4*a*).

Nonetheless, the analogy between ecosystem functioning and Rayleigh–Bénard convection near the bifurcation point, which was claimed to be universal by Schneider & Kay (1994), breaks down when the food webs contain more than one trophic level. Thermodynamically, such more complex food webs behave differently from Rayleigh–Bénard convection near the bifurcation point (figure 4*b*,*c*). In this matter, our analysis shows that one has to make a crucial distinction between the ‘simple’ dissipative systems that are traditionally discussed in connection to dissipative structures, such as Rayleigh–Bénard convection cells near the bifurcation point, and more ‘complex’ dissipative systems, such as ecosystems. This distinction between ‘simple’ and ‘complex’ relates to the number and type of thermodynamic gradients that are exploited. In ‘simple’ dissipative systems, only a single thermodynamic gradient is exploited. This single thermodynamic gradient is the temperature difference *T*_H_ − *T*_L_ in Rayleigh–Bénard convection, and likewise, the difference in chemical potential *μ*_R_ − *μ*_W_ between resource and waste in our ecosystem models. By exploiting this primary thermodynamic gradient, the consumer population can build up biomass. However, by doing so, the consumer biomass also creates a new thermodynamic gradient *μ*_C_ − *μ*_W_, which can now be exploited by a predator at a higher trophic level. Consequently, it is not justified to simply qualify ecosystems and food webs as ‘dissipative structures’ in the sense of Rayleigh–Bénard convection near the bifurcation point. Although each trophic compartment may thermodynamically act as ‘dissipative structure’, the food web as a whole behaves differently, as it actually comprises a hierarchy of interacting ‘dissipative structures’. As a consequence, the thermodynamic response of the food web as a whole will differ from that of a simple ‘dissipative structure’ like Rayleigh–Bénard convection near its bifurcation point. This is the principal reason why the state selection and gradient response principles forwarded by Schneider & Kay (1994) are not generally valid. These principles hold for a single trophic compartment, but are no longer generally true when the food web becomes more complex and more than one trophic level is present.

## 6. Summary: entropy production and ecosystem functioning

Overall, from our analysis, we conclude that Schneider & Kay (1994) have forwarded a too simplistic analogy between the thermodynamic operation of ecosystems and Rayleigh–Bénard convection. The consequence of this is that state selection and gradient response principles are not generally applicable to ecosystems. Because of trophic interactions across more than one level, the stable state of the ecosystem is not necessarily the one that has the highest entropy production rate, thus invalidating the state-selection hypothesis. More generally, this implies that there is no general relation between the stability of the steady state in a nonlinear system and the associated entropy production rate of that state. Therefore, the co-occurrence of stability and MEP, like observed in model simulations of ocean circulation (e.g. Shimokawa & Ozawa 2001), seems to be a coincidental finding rather than an indication of general principle. Similarly, the total entropy production does not necessarily increase when the primary thermodynamic gradient increases, thus invalidating the gradient response hypothesis. From an ecological point of view, this implies that a more complex ecosystem (defined as having more trophic levels) must not necessarily be associated with an increased entropy production rate. However, the hypothesis of Schrödinger (1944), as reformulated and sharpened by Ulanowicz & Hannon (1987), which states that living communities augment the rate of entropy production over what would be found in the absence of biota, holds for all the food webs tested here.
